# A new technique for genome-wide mapping of nucleotide excision repair without immunopurification of damaged DNA

**DOI:** 10.1016/j.jbc.2022.101863

**Published:** 2022-03-23

**Authors:** Sizhong Wu, Yanchao Huang, Christopher P. Selby, Meng Gao, Aziz Sancar, Jinchuan Hu

**Affiliations:** 1 Shanghai Fifth People’s Hospital, Fudan University, and Shanghai Key Laboratory of Medical Epigenetics, International Co-laboratory of Medical Epigenetics and Metabolism (Ministry of Science and Technology), Institutes of Biomedical Sciences, Fudan University, Shanghai, China; 2 Department of Biochemistry and Biophysics, School of Medicine, University of North Carolina, Chapel Hill, North Carolina, USA

**Keywords:** XR-seq, nucleotide excision repair, UV-photoproducts, cyclobutane pyrimidine dimers, excision repair mapping, ATL-XRseq, (6–4)PP, (6–4) pyrimidine–pyrimidone photoproduct, ATL-XR-seq, dA-tailing ligation eXcision Repair sequencing, CPD, cyclobutane pyrimidine dimer, IP, immunoprecipitation, NTS, nontranscribed strand, TCR, transcription-coupled repair, TE, Tris-EDTA, TFIIH, Transcription factor II H, TPL, triptolide, TS, transcribed strand, ss, single-stranded, qPCR, quantitative PCR, XPG, xeroderma pigmentosum complementation group G, XR-seq, eXcision Repair sequencing

## Abstract

Nucleotide excision repair functions to protect genome integrity, and ongoing studies using excision repair sequencing (XR-seq) have contributed to our understanding of how cells prioritize repair across the genome. In this method, the products of excision repair bearing damaged DNA are captured, sequenced, and then mapped genome-wide at single-nucleotide resolution. However, reagent requirements and complex procedures have limited widespread usage of this technique. In addition to the expense of these reagents, it has been hypothesized that the immunoprecipitation step using antibodies directed against damaged DNA may introduce bias in different sequence contexts. Here, we describe a newly developed adaptation called dA-tailing and adaptor ligation (ATL)–XR-seq, a relatively simple XR-seq method that avoids the use of immunoprecipitation targeting damaged DNA. ATL-XR-seq captures repair products by 3′-dA-tailing and 5′-adapter ligation instead of the original 5′- and 3′-dual adapter ligation. This new approach avoids adapter dimer formation during subsequent PCR, omits inefficient and time-consuming purification steps, and is very sensitive. In addition, poly(dA) tail length heterogeneity can serve as a molecular identifier, allowing more repair hotspots to be mapped. Importantly, a comparison of both repair mapping methods showed that no major bias is introduced by the anti-UV damage antibodies used in the original XR-seq procedure. Finally, we also coupled the described dA-tailing approach with quantitative PCR in a new method to quantify repair products. These new methods provide powerful and user-friendly tools to qualitatively and quantitatively measure excision repair.

Nucleotide excision repair is the only DNA repair pathway that removes bulky adducts induced by various environmental carcinogens, including UV, benzopyrene and aflatoxin, and chemotherapeutic drugs such as cisplatin and its derivatives (1, 2). In this pathway, two incisions that bracket the damage are made in the damaged strand, allowing release of a single-stranded (ss) fragment containing the damage (∼12 nt for prokaryotes and ∼26 nt for eukaryotes (1, 3)). The resulting gap is sealed by DNA polymerases and ligases to complete repair (4). The prokaryotic and eukaryotic proteins that catalyze repair perform the same overall function but are not homologous.

While this basal repair reaction occurs throughout the genome, a second transcription-coupled repair (TCR) pathway targets transcription-blocking lesions in the template strand of active genes for more rapid repair (5, 6). TCR was discovered decades ago in both prokaryotes (7) and eukaryotes (8) and is now known to require, in addition to basal repair factors, prokaryotic or eukaryotic transcription-repair coupling factors (5, 6). The discovery of TCR has stimulated interest in the question of repair heterogeneity as a function of DNA metabolism and structure (9, 10, 11), as heterogeneity may impact biological outcomes, including viability and the distribution of mutations which might be related to cancer (12, 13, 14).

Methods to characterize the extent and distribution of repair across the genome have evolved. Early methods utilized measurements of damage, or sequencing approaches, and repair was determined as the loss of damage with time (15, 16, 17, 18, 19, 20, 21, 22). This valuable approach suffers from limited sensitivity especially at early time points or with cells having limited repair capacity, situations in which repair is measured as the difference between large numbers (23). We developed more sensitive methods that measure repair directly based upon capturing and analyzing the excision products generated during repair (24). To quantify excision products, they are isolated from cells, and radiolabeled or biotin-labeled together with an added internal standard oligonucleotide. Samples are then separated with a gel, and the signals are developed (excision assay) (25, 26). To map repair sites across the genome, we developed excision repair sequencing (XR-seq) (27). For this, adapters are ligated to the ends of excision products. Following purification and reversal of the DNA damage, libraries are generated by polymerase chain reaction (PCR) for next-generation sequencing (28).

To date, valuable results have been obtained with these methods, confirming the *in vitro* identified dual incision patterns (1) and demonstrating in exquisite detail the extent of TCR *in vivo*. Moreover, XR-seq has been applied to the repair of damage induced by UV (19), cisplatin (29) and benzopyrene (30) in multiple species and organs, including human and lemur cells (28), *Arabidopsis* (31, 32), *Escherichia coli* (33), yeast (34), *Drosophila* (35), and mouse tissues (36). Experiments employing XR-seq have revealed interesting aspects of repair regulation by transcription, transcription factor binding, replication, chromatin states, circadian rhythm, and other factors and how repair patterns relate to the distribution of mutations in cancer genomes (12, 37, 38, 39, 40).

Although the excision assay and XR-seq are specific and robust, technical factors limit their application. For the excision assay, there is the use of radioactivity or expensive detection reagents (25). XR-seq is laborious and time-consuming (41), and has relatively low yield (42), as it involves two immunoprecipitation (IP) steps that require expensive antibodies, and of considerable concern here, the anti-DNA damage antibodies may prefer certain underlying sequences and/or surrounding nucleotides and introduce a sequence bias into the final results (17).

Here, we designed a new strategy based on the 3′ dA-tailing and 5′ adapter ligation (ATL) reactions to allow PCR amplification to produce sequencing libraries (ATL-XR-seq) and quantitative PCR (qPCR) to quantify excised oligomers (ATL-XR-qPCR). The 3′ dA-tailing followed by 5′ adapter ligation instead of simultaneous ligation of both 5′ and 3′ adapters eliminates adapter dimers and obviates the anti-damage IP and gel purification steps to increase yield and save time and money. Comparison of the two mapping methods revealed no major bias introduced by the use of anti-damage antibodies in XR-seq. We find ATL-XR-seq and ATL-XR–qPCR to be sensitive and easy-to-use tools to measure nucleotide excision repair qualitatively and quantitatively.

## Results

### Development of dA-tailing and ligation-mediated XR-seq (ATL-XR-seq)

**Figure 1:**
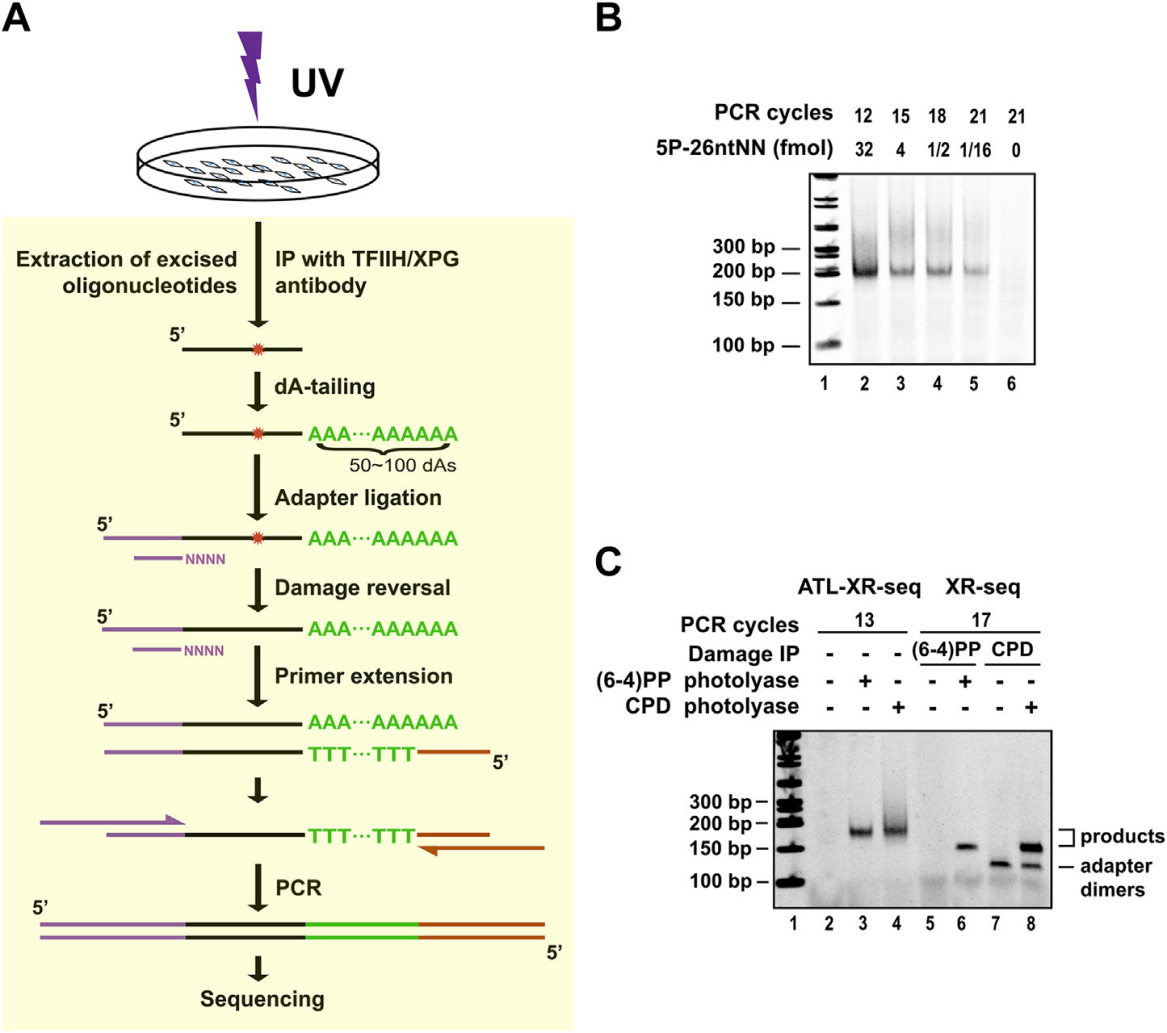
**ATL-XR-seq method.***A*, schematic of ATL-XR-seq following UV. Following appropriate repair times, cell extracts are prepared. Primary excision products are then purified by co-IP with TFIIH (XPB or p62) or XPG antibodies. Then, a poly(dA) tail is added to the 3′ end by terminal transferase. The reaction product is then ligated to an adapter at its 5′ end. The UV photoproducts are repaired using either (6–4)PP photolyase or CPD photolyase, then a ss oligo with 30-dT residues at the 3′ end and a 5′ handle is annealed and extended by DNA polymerase. The full-length extension products possess the original excision product sequences (in antisense form) located between 3′ and 5′ handles. The extension products are then amplified by PCR to generate libraries for high-throughput sequencing. *B*, quality check of ATL-XR-seq libraries made from a 26-nt mimic excision product (5P-26 nt NN). *C*, quality check of representative ATL-XR-seq and XR-seq libraries. About 3.5 million [for (6–4)PP] or 50 million (for CPD) cells were used for each sample. The PCR products were separated by 5% native polyacrylamide gel electrophoresis. The bands corresponding to “adapter dimers” were only seen in XR-seq of CPD but not (6–4)PP since more adapters were used for the CPD sample (see “Experimental procedures”). Adapter dimers are commonly seen with both photoproducts and may be the predominant PCR product under conditions in which the yield of excision product is poor, for example, early time points, repair mutants, and certain tissues and species. (6–4)PP, (6–4) pyrimidine–pyrimidone photoproduct, ATL-XR-seq; dA-tailing ligation eXcision Repair sequencing; CPD, cyclobutane pyrimidine dimer; IP, immunoprecipitation; TFIIH, Transcription factor II H; XPB, xeroderma pigmentosum complementation group B; XPG, xeroderma pigmentosum complementation group G.

Nucleotide excision repair products are short, approximately 24- to 28-nt ss oligonucleotides containing the damage (24). To capture these products from cell extracts for mapping repair (ATL-XR-seq and XR-seq schemes are shown in Figs. 1*A* and S1), two IP methods are available. Following excision, excision products remain bound to repair factors transcription factor II H (TFIIH) and xeroderma pigmentosum complementation group G (XPG), and these bound products can be precipitated using anti-TFIIH or anti-XPG antibodies. Alternatively, excision products may be precipitated directly using anti–cyclobutane pyrimidine dimer (CPD)–DNA or anti–(6–4) pyrimidine–pyrimidone photoproduct [(6–4)PP]–DNA antibodies. Either immunopurification method may be used in the initial step of XR-seq, while immunopurification with anti-TFIIH or anti-XPG antibodies is used in ATL-XR-seq.

The original XR-seq method includes ligation of double-stranded adapters to the 5′ and 3′ ends of each immunoprecipitated excision product. Unfortunately, with this approach, a large number of adapter dimers are produced during a later PCR step, especially under suboptimal repair conditions which entail a low amount of excision. This limits the flexibility of XR-seq (27) and makes necessary a second IP step with an anti-damage antibody, which introduces a possible bias associated with antibody specificity.

In order to overcome these shortcomings, we envisaged the ATL-XR-seq strategy that employs only one adapter. The idea is to use terminal transferase to add a poly(dA) “tail” to the 3′ end of excision products. These tails could be subsequently annealed to a primer with 30 dTs at the 3′ end as a 3′ handle. In ATL-XR-seq (Figs. 1*A* and S1), the dA-tailed excision product is then ligated to a single adapter at the 5′ end, repaired with the appropriate photolyase, and the 30 dT-containing primer is annealed and extended by DNA polymerase. The extension product possesses the excision product sequence in antisense orientation flanked by 5′ and 3′ handles and is amplified by PCR to generate libraries for next-generation sequencing.

To develop ATL-XR-seq, we initially focused on the novel dA-tailing step and subsequent dT-oligo annealing and extension. Addition of dNTPs to the 3′ end of ssDNA by terminal transferase is known to vary as a function of reaction conditions. In a pilot experiment, we employed a 26-nt undamaged oligo (5FAM-26 nt) as a substrate for dA tailing by terminal transferase and saw various levels of tailing under the conditions employed (Fig. S2*A*). We utilized conditions that produced robust tailing and attempted the ATL-XR-seq procedure, again using the 26-nt undamaged oligo (5P-26-nt NN) as a mock excision product, and using a primer with a run of 30 T residues at the 3′ end (“30T-Primer”) for primer extension. As shown in Figure 1*B*, the procedure successfully produced potential sequencing libraries from a wide range of input DNA concentrations with no apparent adapter dimers. The ATL-XR-seq procedure was then applied to characterize excision products from UV-irradiated HeLa cells. Fig. S2, *B* and *C* show the length of the poly(dA) tails in the sequencing libraries generated for CPD and (6–4)PP repair. The peak length was around 30 nt.

To directly compare library generation by ATL-XR-seq and XR-seq, both methods were performed to characterize repair in UV-irradiated HeLa cells. For each damage type, the excision products were purified by co-IP with anti-XPG antibody and equally divided into two portions, for processing by old and new methods. Figure 1*C* indicates that both methods produced enough PCR products for sequencing. The band representing the ATL-XR-seq library is slightly larger due to the presence of the poly(dA) tails and the corresponding adapter poly(dT) tail. Notably, ATL-XR-seq required fewer PCR cycles than XR-seq, indicating a much higher yield by ATL-XR-seq. Moreover, due to the relatively low abundance of CPD excision products, the CPD library of XR-seq had an obvious band of adapter dimers, while the new method did not. Therefore, the ATL-XR-seq method has no need for gel purification which is necessary in XR-seq.

### Comparison of data from XR-seq and ATL-XR-seq

**Figure 2:**
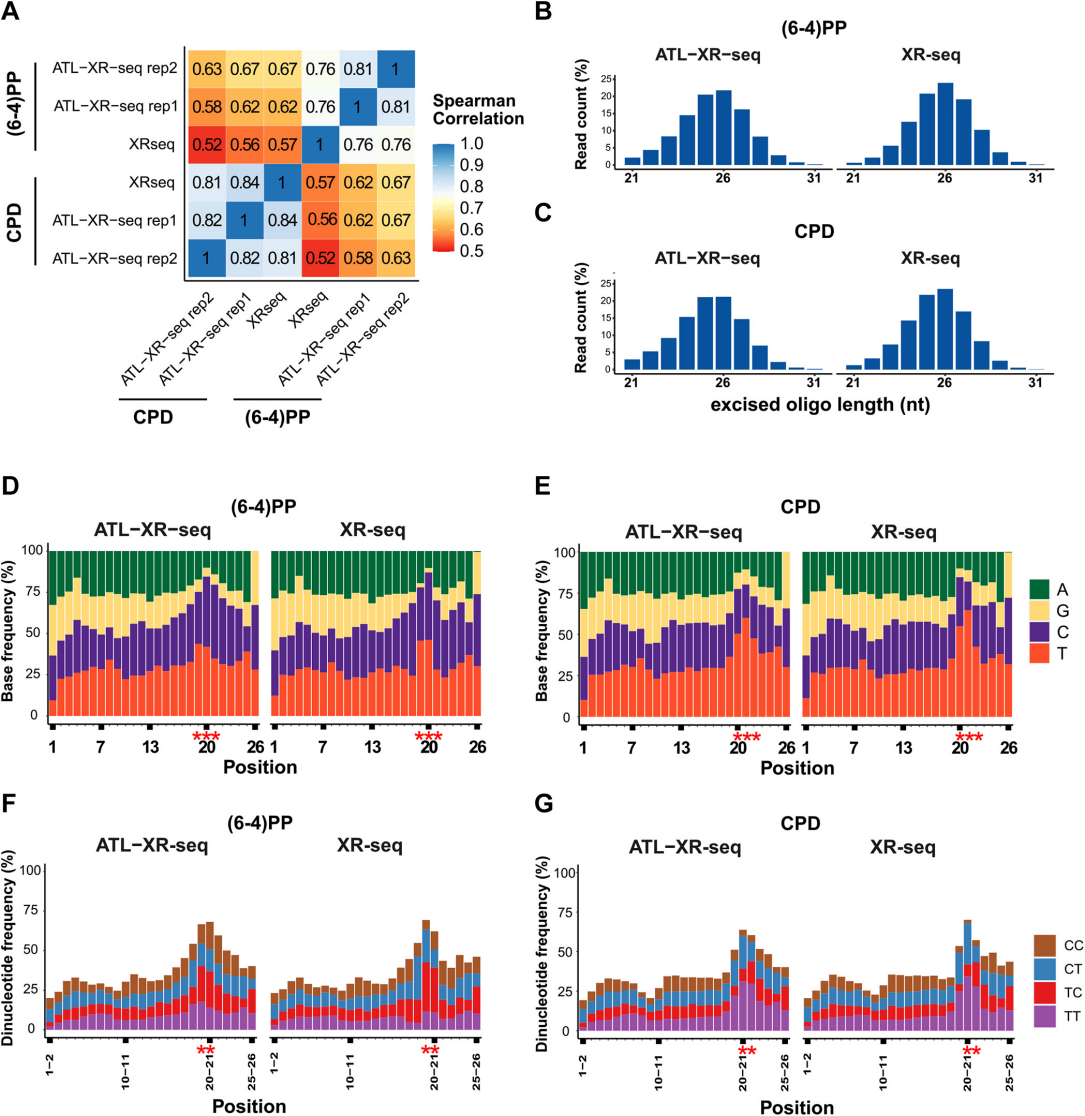
**Comparison of ATL-XR-seq and XR-seq.***A*, Spearman’s correlation coefficient among all samples. *B* and *C*, length distribution of primary excision products in (6–4)PP (*B*) and CPD (*C*) by ATL-XR-seq (*left*) and XR-seq (*right*). *D* and *E*, nucleotide distribution along 26-nt sequencing reads of (6–4)PP (*D*) and CPD (*E*) by ATL-XR-seq (*left*) and XR-seq (*right*). *F* and *G*, dipyrimidine frequency of (6–4)PP (*F*) and CPD (*G*) along 26-nt sequencing reads by ATL-XR-seq (*left*) and XR-seq (*right*). *Red stars* represent potential damage sites with higher frequency of pyrimidines (*D* and *E*) or dipyrimidines (*F* and *G*) than other sites. Replicate 1 of ATL-XR-seq is shown in *B–G*. (6–4)PP, (6–4) pyrimidine–pyrimidone photoproduct, ATL-XR-seq; dA-tailing ligation eXcision Repair sequencing; CPD, cyclobutane pyrimidine dimer; XR-seq, eXcision Repair sequencing.

Using UV-irradiated HeLa cells, we conducted two biological replicates for ATL-XR-seq and one for XR-seq as described previously. In general, the data for each damage type were similar regardless of the method, and both methods detected differences in repair of (6–4)PPs *versus* CPDs (Fig. 2*A*). Read length distributions of excision products were similar for both damages and both methods, with the peak at 25 to 26 nt (Figs. 2, *B* and *C*, S3, *A* and *D*), even though ATL-XR-seq did not employ gel purification, which could potentially influence the size of products recovered. Regarding the length of the excision products detected, it is worth noting that by the method employed, when the poly(dA) tails from ATL-XR-seq reads were trimmed, any real dA(s) at 3′ end would also be removed. This would not alter the repair pattern across the genome. However, it will change the nucleotide distribution at the 3′ end and slightly shorten the average length of reads and the distance of lesions to the 3′ end. Therefore, for Figure 2, *B* and *C*, we manually removed dA(s) at the 3′ end of XR-seq–derived excision reads for a fair comparison between the two methods.

We then compared frequency distribution profiles for single nucleotides and dipyrimidines along 26-nt reads for each damage and method. Since the repair enzyme incises the substrate at approximately 6 nt 3′ to the damage and 19 nt 5′ to the damage, an elevated frequency of pyrimidine residues is expected at the corresponding location in excision products. Figure 2, *D–G* shows that the distribution of nucleotides (*D*, *E*) and dipyrimidines (*F*, *G*) for each damage type were similar in the old and new methods. For (6–4)PP, TC and TT were the predominant, presumptive damage sites which were located at about 5 to 6 nt from the 3′ end (Figs. 2*F* and S3*B*). For CPD, the major presumptive damage site was TT, which was located about 4 to 5 nt from the 3′ end (Figs. 2*G* and S3*E*).

**Figure 3:**
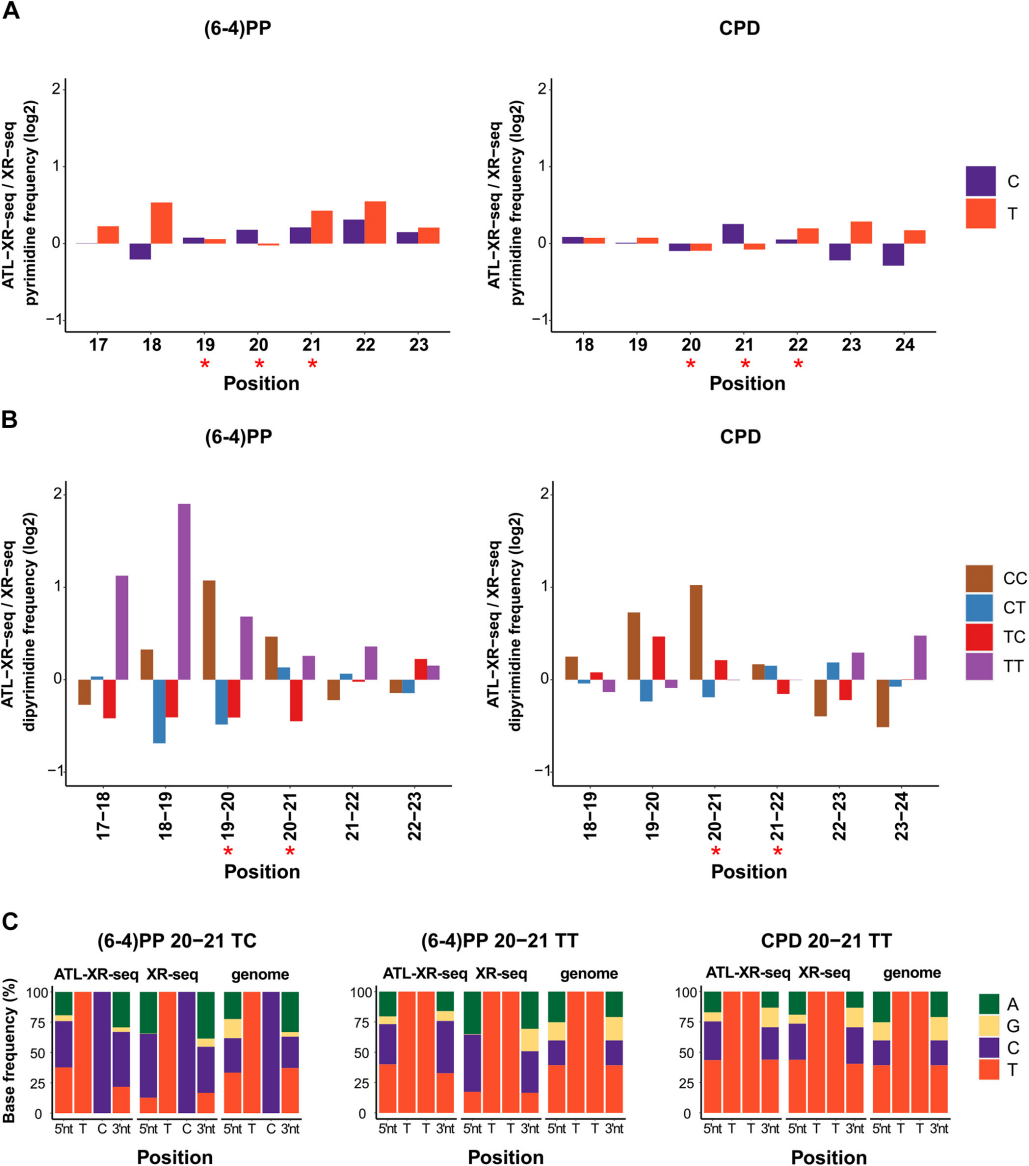
**Excision product sequence differences between ATL-XR-seq and XR-seq.***A*, comparison of pyrimidine (T and C) frequencies (rates) along (6–4)PP (*left*) and CPD (*right*) 26-nt sequencing reads obtained by old and new methods. *B*, comparison of dipyrimidine (TT, TC, CT, CC) frequencies along (6–4)PP (*left*) and CPD (*right*) 26-nt sequencing reads obtained by old and new methods. *Red stars* represent potential damage sites in (*A*) and (*B*). *C*, nucleotide distribution at positions adjacent to potential damage sites (TC [*left*] and TT [*middle*] for (6–4)PP and TT [*right*] for CPD, each at 20–21 nt from the 5′ end). Random TC and TT from the genome were presented as control. Data from replicate 1 of ATL-XR-seq is used. (6–4)PP, (6–4) pyrimidine–pyrimidone photoproduct, ATL-XR-seq; dA-tailing ligation eXcision Repair sequencing; CPD, cyclobutane pyrimidine dimer; XR-seq, eXcision Repair sequencing.

It was reported that the anti-DNA damage–specific antibodies used have preferred binding sequences (17) which might cause bias in not only XR-seq but also other antibody-based sequencing methods (*e.g.*, Damage-seq (19)). The results in Figure 2 do not reveal any major antibody-associated bias. To examine possible bias in more detail, we first compared the pyrimidine composition of presumptive damage sites obtained by XR-seq and ATL-XR-seq. As shown in Figure 3*A*, the ratios of T and C at presumptive damage sites (positions 19–21), as measured by the two methods, were minor. We also inspected relative changes in dipyrimidine frequencies (Fig. 3*B*). For both damages, the ratio of CC increased at potential damage sites in ATL-XR-seq, consistent with the fact that both antibodies have low affinity to the relatively uncommon CC photoproducts. However, the ratio of TT which has relatively high affinity toward both antibodies either had no obvious change (CPD) or slightly increased [for (6–4)PP] at potential damage sites when detected by ATL-XR-seq. This slight increase is opposite to the expectation which follows from the relatively high affinity of the antibodies to TT photoproducts. This modest effect may be due in part to the selection of surrounding nucleotides by the antibody and different nucleotide frequencies at the 3′ next to TT and TC in the human genome (see later).

Examining pyrimidine compositions adjacent to potential (6–4)PP, we again found minor differences in nucleotide frequencies between the two methods (Fig. 3*A*). To examine these differences in more detail, we extracted reads containing the dominant damage sequences [TT and TC for (6–4)PP and TT for CPD] at potential damage sites and compared the frequencies of neighboring nucleotides. As shown in Figure 3*C*, at the 5′ upstream position of both (6–4)PPs, the ratio of T and G increased in ATL-XR-seq compared to XR-seq, whereas the other two nucleotides decreased in the new method. At the 3′ downstream position, the pyrimidines increased, while the purines declined in ATL-XR-seq. Consistent with previous data (27), this result indicates that the (6–4)PP antibody prefers “A” at 3′ neighbor position. Notably, the ratio of A at 3′ adjacent to TC is higher than that to TT in the human genome (Fig. 3*C*), so the antibody might selectively enrich TC for human genome samples despite the fact that it has a higher affinity to TT. In contrast, the nucleotide ratio at both sides of TT–CPD had no apparent difference between the two methods. As noted previously, antibody bias may be associated with these minor sequence effects on measurement of (6–4)PP repair.

### Exploring transcription coupled repair by ATL-XR-seq

**Figure 4:**
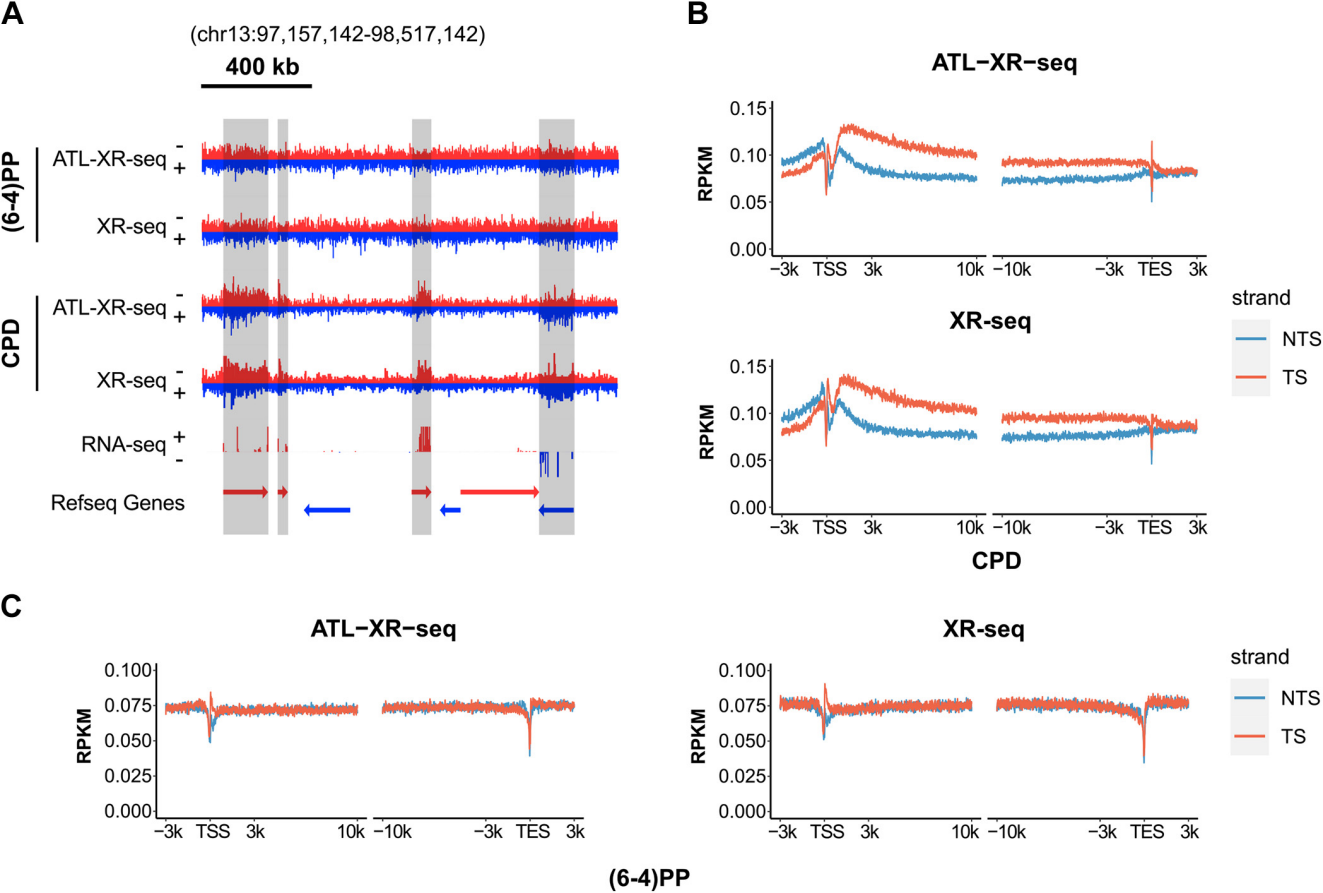
**Repair pattern along genes by ATL-XR-seq and XR-seq.***A*, screenshot of stranded (6–4)PP and CPD repair by ATL-XR-seq and XR-seq in a 1.36-Mb region of chromosome 13. Total stranded RNA-seq of HeLa-S3 cells from ENCODE and Refseq Genes from NCBI are plotted at the bottom. The shadows highlight transcribed genes. *B* and *C*, metaprofiles of CPD (*B*) and (6–4)PP (*C*) repair patterns around TSSs and TESs of annotated genes more than 12 kb in length (16,366 genes). Replicate 1 of ATL-XR-seq is shown. (6–4)PP, (6–4) pyrimidine–pyrimidone photoproduct, ATL-XR-seq; dA-tailing ligation eXcision Repair sequencing; CPD, cyclobutane pyrimidine dimer; TES, transcription end site; TSS, transcription start site; XR-seq, eXcision Repair sequencing.

XR-seq is a powerful tool for investigating strand-specific TCR (27), and we were interested in comparing TCR by the two methods. The screenshots in Figures 4*A* and S4*A* show that by both methods, the template strands (TSs) and nontemplate strands (NTSs) had comparable (6–4)PP repair signals, whereas there was obviously more CPD repair of the TS than the NTS. Metagene analysis indicated that both methods obtained similar overall strand-specific distribution of repair across genes and confirmed the conclusion that TCR had a measurable contribution toward the repair of CPDs (Figs. 4*B* and S4*B*) but not (6–4)PPs (Figs. 4*C* and S4*C*) under our experimental conditions. Interestingly, for CPD repair, ATL-XR-seq showed a sharp peak in the TS around the transcription end site, whereas the TS peak near the transcription start site was higher in XR-seq. This discrepancy might be attributed to the antibody-related sequence bias and the uneven sequences around the transcription start site and transcription end site.

### Repair hotspots identified by ATL-XR-seq using poly(dA) as an identifier

The dA-tailing step of ATL-XR-seq adds a heterogeneous number of dAs to the 3′ end of excision products. The different lengths of poly(dA) tails in the sequencing reads, which peaked at 30 nt (Fig. S2, *B* and *C*), provide a simple way to distinguish genuine repair hotspots and redundant reads generated by PCR. For the XR-seq method (deduplication by command “Sambamba Markdup”, named “Markdup”), if two or more excised oligomers were identical, they were considered as PCR-generated duplicates and only one would be kept. With XR-seq, multiple repair events at a single damage site were identified as true reads only if they had nonidentical 5′ and/or 3′ ends. For ATL-XR-seq, using poly(dA) as a molecular identifier (named “A_n_-identifier”), reads that are identical but that have different poly(dA)-tail lengths are retained for analysis. Since ATL-XR-seq produced more reads than XR-seq, we used poly(dA)-trimmed ATL-XR-seq data to mimic XR-seq data and compared the two deduplication methods.

**Figure 5:**
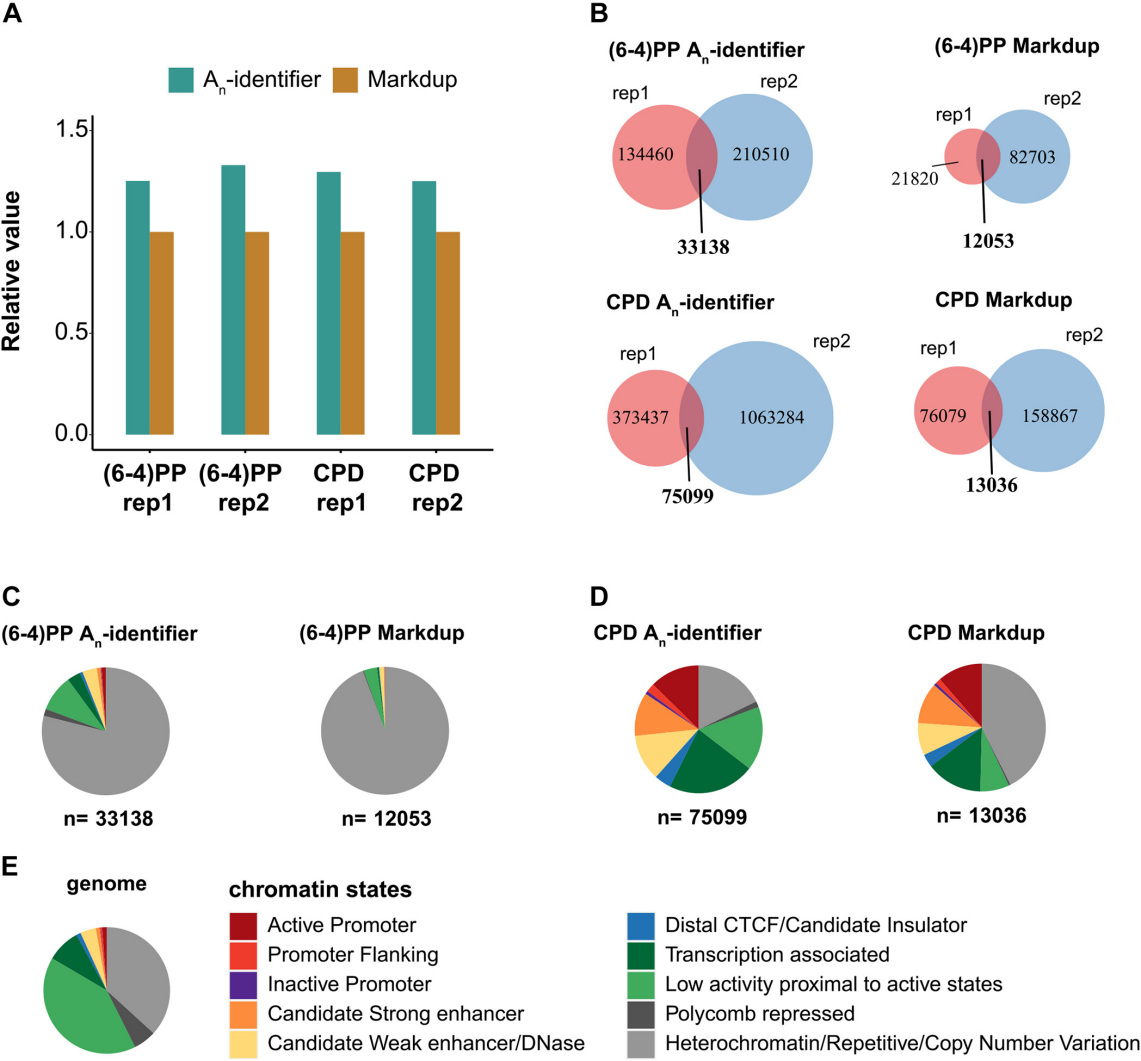
**Identification of repair hotspots using the poly(dA) as an identifier.***A*, comparison of the numbers of deduplicated reads using “A_n_-identifier” and “Markdup” for deduplication. *B*, repair hotspots of (6–4)PP and CPD by two deduplication methods. The overlapping hotspots between two replicates are considered as “confident repair hotspots.” The numbers of hotspots vary between biological replicates as they have different numbers of total qualified reads (see “Experimental procedures”). *C–E*, distribution of (6–4)PP (*C*) or CPD (*D*) confident repair hotspots and reference genome (*E*) among 10 chromatin states. (6–4)PP, (6–4) pyrimidine–pyrimidone photoproduct, CPD, cyclobutane pyrimidine dimer.

First, the A_n_-identifier method retrieved ∼30% more reads than Markdup using the same dataset (Fig. 5*A*), indicating that many repair hotspots were discarded by the old method. Indeed, the new method identified many more hotspots than the old method for all samples (Fig. 5*B*). For both methods, a significant portion of hotspots from two biological replicates overlapped and were considered as “confident repair hotspots” (Fig. 5*B*). The distribution of these confident repair hotspots among different chromatin states was analyzed (Fig. 5, *C–E*). Intriguingly, for both damage types, the repetitive regions occupied a larger portion for the confident repair hotspots identified by Markdup than those identified by the new method, suggesting that the new method retains a greater diversity of hotspots than the old method (Fig. 5, *C* and *D*). Compared to reference genome (Fig. 5*E*), confident repair hotspots identified by the new method were highly enriched in the heterochromatin/repetitive/copy number variation regions for (6–4)PP (Fig. 5*C* left) and open chromatin regions (active promoter, candidate strong enhancer, etc.) for CPD (Fig. 5*D* left). A possible explanation is that (6–4)PP is repaired much faster than CPD, so at the time of sampling, (6–4)PPs in regions with most open chromatin were largely removed, while CPDs in these regions were being repaired (9).

### Quantification of global excision repair by ATL-XR-qPCR

**Figure 6:**
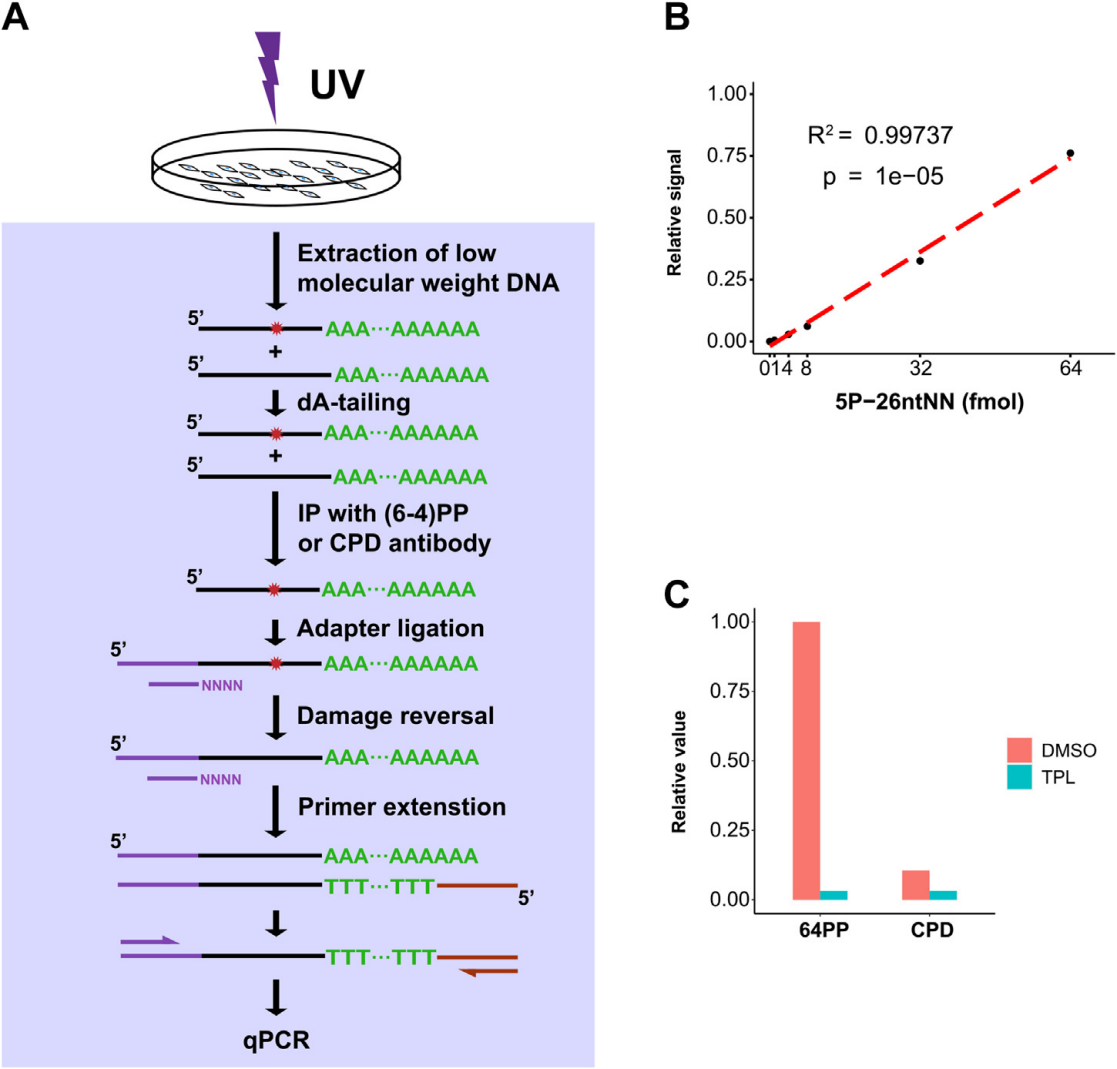
**ATL-XR-qPCR method.***A*, schematic of ATL-XR-qPCR. The asterisk indicates UV-induced (6–4)PP or CPD. *B*, standard curve showing ATL-XR-qPCR of 26-nt mimic excision product (5P-26 nt NN). *C*, quantification of (6–4)PP and CPD repair products by ATL-XR-qPCR. Repair was performed in the absence or presence of TFIIH inhibitor TPL (1 μM). Repair products were normalized to (6–4)PP products in the absence of TPL. Replicate 1 of ATL-XR-qPCR is shown in (*C*). (6–4)PP, (6–4) pyrimidine–pyrimidone photoproduct, ATL-XR-seq; dA-tailing ligation eXcision Repair sequencing; CPD, cyclobutane pyrimidine dimer; TFIIH, Transcription factor II H; TPL, triptolide; qPCR, quantitative PCR.

The excision assay has been used as a complement to XR-seq to quantify excision products. In this assay, isolated excision products are labeled and resolved on a sequencing gel to determine the length distribution of the excision products and to compare the amount of excision either with standards or between different samples (24, 25, 26). Based upon the success of ATL-XR-seq, we developed an alternative method to quantify excision that does not rely on the labeling and detection methods of the excision assay. Instead, detection is by qPCR. To avoid variations caused by operation and obtain results comparable to the excision assay, the workflow of ATL-XR-qPCR is adapted from both excision assay and ATL-XR-seq, as shown in Figure 6*A*. The first step is isolation of low-molecular-weight DNA from cells, as is done in the excision assay. In ATL-XR-qPCR, the next step is dA tailing, followed by IP with anti-(6–4)PP or anti-CPD antibodies. Then an adapter is ligated to the 5′ end, damage is reversed with the appropriate photolyase, and the primer is extended as in ATL-XR-seq. The extension product is then subject to qPCR. A standard curve with the 26-nt mimic excision products showed a linear correlation between the amounts of input and signal across a wide range, indicating the feasibility of this method (Fig. 6*B*). The ATL-XR-qPCR assay was used to determine the effect of an xeroderma pigmentosum complementation group B inhibitor, triptolide (TPL) (43) on nucleotide excision repair. As shown in Figures. 6*C* and S5*A*, the repair of (6–4)PP was much stronger than that of CPD, and both were greatly repressed by TPL, indicating the utility of the ATL-XR-qPCR assay.

## Discussion

XR-seq has been very useful for studying genome-wide nucleotide excision repair and has been applied to numerous species and multiple damage types (41) to learn how repair is prioritized in cells. However, considerable investments of time and resources are required to apply the method, and XR-seq has not enjoyed widespread use. The key improvement of ATL-XR-seq is using dA tailing followed by one-side ligation instead of simultaneous ligation on both sides of excision products, which eliminates adapter dimers and obviates the need for anti-DNA damage antibody IP and gel purification steps. Our data indicate that ATL-XR-seq produces nearly identical repair maps as XR-seq with fewer PCR cycles and shorter handling time. Moreover, this improvement is compatible with all previous variations of XR-seq (*e.g.*, XR-seq for cisplatin). Notably, for damage such as benzopyrene, which cannot be reversed, translesion XR-seq was developed (29, 30, 32, 41). Translesion XR-seq employs a DNA polymerase capable of bypassing the damage to create an antisense strand which can be amplified to generate a library (30). In the case of translesion ATL-XR-seq, a translesion DNA polymerase may be used in the primer extension step. In conclusion, ATL-XR-seq is a very promising method for excision repair mapping. However, since there is no DNA damage–specific purification after dA tailing and 5′ adapter ligation, any nonspecific DNA fragments remaining after the IP step as well as any DNA contaminating the reagents can also be captured and amplified. Therefore, high-quality reagents are especially important for ATL-XR-seq. Moreover, ATL-XR-seq might not be applicable for cells containing significant amounts of short DNA fragments, for example, those undergoing apoptosis. Similar to the original XR-seq, an additional IP step with anti-DNA damage antibody after ligation can resolve this issue. Thanks to the omission of gel purification, this modified ATL-XR-seq retains the advantage of simple operation and high yield over the original XR-seq.

Sequence preference is an inevitable property of anti-DNA damage antibodies which might cause problems in some applications, for example, comparing the spectra of damage and mutation in cancer genomes. Damage-seq is an antibody-based method that can detect bulky adducts including UV-induced CPDs and (6–4)PPs at single-nucleotide resolution (19). The predominant CPD sequence revealed by Damage-seq was TT, followed by CT, whereas TC and CC were minor. However, CPD-seq, another single-nucleotide sequencing method based on the CPD-specific endonuclease T4 pyrimidine dimer glycosylase, revealed a smaller percentage of TT at CPD sites, which was followed by TC, CT, and CC (44). The total ratio of C-containing CPD was much greater in CPD-seq than that in Damage-seq. Since the commercial CPD antibody (TDM-2) had higher affinity toward TT-CPD over other sequences (17), it was claimed that CPD-seq should reflect the actual CPD composition with less bias and was more appropriate for comparisons of the distribution of mutations in skin cancers (44). Intriguingly, the bias caused by CPD antibody can be evaluated by the comparison between ATL-XR-seq and XR-seq since only XR-seq includes the damage IP step. Surprisingly, the proportion of TT excision products at potential CPD damage sites was virtually unchanged between the two methods. The relative ratio of CC increased in ATL-XR-seq, although the relative amount of CC is modest compared with the other dipyrimidines. This result indicates that the antibody exerted a modest impact on the detection of CPD sequences, and CPD-seq seemed to cause a stronger sequence bias than Damage-seq probably due to the sequence preference of T4 pyrimidine dimer glycosylase.

On the other hand, some variability in (6–4)PP sequences repaired was evident between XR-seq and ATL-XR-seq. Consistent with our previous data (27), XR-seq identified CTCA (and CTTA) as the preferred (6–4)PP sequence. Interestingly, this feature was largely absent in ATL-XR-seq. On face value, it appears that the XR-seq identified preference for C 5′ and A 3′ to the putative repaired (6–4)PP is an artifact introduced by the antibody. However, an independent study examined sequences at sites of (6–4)PP damage formation *in vivo*. This study, which did not use antibodies, found enrichment of A 3′ to (6–4)PPs induced in irradiated DNA (45), partially reconstructing the bias seen in XR-seq repair products. Therefore, more investigation is needed to resolve this point of departure between XR-seq and ATL-XR-seq.

Recently, it was reported that nucleotide excision repair exhibited hotspots (42), especially at early time points. However, the deduplication step of XR-seq data processing eliminates identical reads, so as to avoid including artifactual duplications produced by PCR. The only way that multiple excision products originating from a single damage site are considered true reads is if the excision products detected have different ends. This analysis may discard true duplicates and under-represent hotspots. Adding a random barcode (unique molecular identifier) to the adapter could solve this issue; however, the barcoded adapter would be very long and might cause unspecific PCR products (46). In contrast, the poly(dA) tails of ATL-XR-seq can serve as a unique molecular identifier and thus identify more repair hotspots, as shown in this investigation.

Measuring excision products is a sensitive and direct way to quantify ongoing nucleotide excision repair. Radioactive labeling, especially 3′ end labeling with α-^32^P cordycepin has provided unrivaled sensitivity to detect less than 1 fmol of excision products from more than 10 million cells (24, 27). However, not all labs are able to perform radioactive experiments, and this expensive reagent has become unavailable. Using another radioactive nucleotide such as α-^32^P dATP or γ-^32^P ATP again involves use of isotopes and could compromise the resolution and/or signal-to-noise ratio. Biotin-labeled dUTP or ddUTP may be used but with reduced sensitivity, and the procedure is more complicated (additional membrane transferring and blotting steps are required) (25, 26). Here, we present the ATL-XR-qPCR assay to measure excision products with common equipment and reagents. The data from control, undamaged mimic oligonucleotide indicate that ATL-XR-qPCR is able to detect less than 5 fmol of excision product (Fig. 6*B*). This sensitivity is higher than that of the biotin-labeling method and close to radioactive-labeling methods, especially when α-^32^P cordycepin is not available. The limitation of ATL-XR-qPCR is that it does not reveal the length distribution of excision products. Nonetheless, ATL-XR-qPCR is sufficient for checking whether the activity of nucleotide excision repair is affected by genetic manipulation or drug treatment.

In summary, ATL-XR-seq has many advantages over the original XR-seq method. It will enable the measurement of difficult samples, such as very few cells, early or late time points, cells with weak repair capacity, and damage for which there is no specific antibody. Furthermore, both ATL-XR-seq and ATL-XR-qPCR are easier to perform than the original methods and therefore may find widespread use.

## Experimental procedures

### Cell lines and UV irradiation

The HeLa-S3 cell line (purchased from American Type Culture Collection) was cultured in standard Dulbecco’s modified Eagle’s medium (Gibco) supplemented with 10% fetal bovine serum (Gibco) and 1% penicillin–streptomycin (Gibco). Cells were incubated at 37 °C in a 5% CO_2_ humidified chamber.

Cells at about 80% confluence were irradiated using a UVC lamp (GE) connected to a digital timer for 10 s (2 J/m^2^/sec) after removing the media. After irradiation, culture medium was added back and cells were incubated at 37 °C in a 5% CO_2_ humidified chamber for the indicated times. After repair, cells were put on ice and washed with ice-cold PBS, then harvested in cold PBS, and collected by centrifugation at 1000*g* at 4 °C for 5 min. For TPL (MedChemExpress) treatment, TPL was added to the medium 10 min prior to UV irradiation and maintained after UV irradiation at a final concentration of 1 μM.

### Oligonucleotides and adapters

The adapter Ad2-ATL was prepared by annealing the following two oligonucleotides: AD2B-ATL (5′-ACACTCT TTCCCTACACGACGCTCTTCCGATCT-3′) and AD2T-ATL (5′-NNNNNAGATCGGAA-SpC3-3′). The primer for extension was 30T-Primer (5′-GACTGGAGTTCAGACGT GTGCTCTTCCGATC[T_30_]-3’) with 30 dTs at 3′ end. The two qPCR primers were qPCR-F (5′-ACACTCTTTCCCTACACGACG-3′) and qPCR-R (5′-TGACTGGAGTTCAGAC GTGT-3′). The oligonucleotides used in control experiments to mimic excision products were 5P-26ntNN (5′-phos-NNCCCGGTTTCTATAAATTGAGCCNN-3′, 5′ phosphorylated for library preparation and qPCR experiments) and 5FAM-26 nt (5′-FAM-CCCCCGGTTTCTATAAATTGAGC CNN-3′, 5′ FAM labeled for fluorescence imaging, used in dA-tailing titration). NEBNext Multiplex Oligos for Illumina (dual index) (New England Biolabs) were used for ATL-XR-seq library preparation. All oligonucleotides used in XR-seq were described before (41).

### Purification of primary excision products by co-IP with anti-XPG antibody

This step is performed as previously described (27). Briefly, at repair times of 30 min [for (6–4)PP] or 1 h (for CPD) following UV irradiation, cells (each 2 × 10^7^ cells) were harvested and resuspended in 800 μl of buffer A (25 mM Hepes pH 7.9, 100 mM KCl, 12 mM MgCl_2_, 0.5 mM EDTA at pH 8.0, 2 mM DTT, 12.5% glycerol, 0.5% Igepal CA-630), followed by incubation on ice for 15 min. These repair times were selected because they have been found to reproducibly produce libraries of acceptable quality by XR-seq. Resuspended cells were transferred to a Dounce homogenizer and stroked 50 to 80 times using a tight plunger. Lysed cells were then centrifuged at 16,850*g* for 30 min at 4 °C to remove the chromatin fraction. Supernatants were transferred to a new tube, and 4 μg of anti-XPG antibody (Santa Cruz Biotechnology, clone 8H7) was added. Of note, TFIIH, XPG, and primary excision products form a tight complex after dual incisions, and both TFIIH and XPG antibodies can efficiently pull down the primary excision products (24). Thus, any TFIIH or XPG antibody can be used if it works for IP. Samples were rotated for 3 to 4 h at 4 °C and then incubated overnight with 25 μl of recombinant protein A/G Plus-agarose (Santa Cruz Biotechnology). The agarose was washed twice with 1 ml of buffer A and twice with 1 ml of buffer B (buffer A plus 0.5% Igepal CA-630), and then DNA was eluted by incubation with 50 μl of elution buffer I (10 mM Tris-Cl pH 8.0, 1 mM EDTA at pH 8.0, 1% SDS) at 65 °C for 5 min. Eluted samples were incubated with 2 μl of proteinase K (New England Biolabs) for 20 min at 55 °C, followed by phenol–chloroform extraction and ethanol precipitation. Purified DNAs were further cleaned through ProbeQuant G-50 Micro Columns (Cytiva) and concentrated by ethanol precipitation. Purified primary excision products were equally divided into two portions for XR-seq and ATL-XR-seq assays.

### ATL-XR-seq

Purified primary excision products were incubated with 0.5 μl of terminal deoxynucleotidyl transferase (New England Biolabs), 7.5 μl of 10 μM dATP, 5 μl of 10 × reaction buffer, and 5 μl of 2.5 mM CoCl_2_ in a total volume of 50 μl at 37 °C for 90 min. The enzyme was inactivated by incubation at 75 °C for 20 min, followed by ethanol precipitation. The DNA pellet was dissolved in 6 μl of 0.1 × Tris-EDTA (TE) buffer (1 mM Tris-Cl pH 8.0, 0.1 mM EDTA) and incubated with 1.5 μl of 10 μM Ad2-ATL and 7.5 μl of 2 × Instant Sticky-end Ligase Master Mix (New England Biolabs) at 4 °C overnight for adapter ligation. After phenol–chloroform extraction and ethanol precipitation, the lesions in ligation products were reversed by CPD or (6–4)PP photolyase as described before (41). Repaired samples were purified by phenol–chloroform extraction and ethanol precipitation, and the DNA pellets were dissolved in 6 μl of 0.1 × TE buffer. DNA samples were then incubated with 1.5 μl of 10 μM primer 30T-Primer and 7.5 μl of Q5 2 × Master Mix (New England Biolabs) for 1 min at 98 °C, annealed for 30 s at 55 °C, and then extended for 90 s at 68 °C. Samples were then incubated with 1 μl of exonuclease I (New England Biolabs) for 15 min at 37 °C to remove excess primers, and then the enzyme was inactivated for 15 min at 80 °C. Three percent of each sample and unrepaired control were used for quality check. The rest of the sample (15 μl) was supplemented with 18 μl of NEBNext Ultra II Q5 Master Mix (New England Biolabs), 5 μl of each primer (10 μM), and 7 μl of water and then amplified in a thermocycler with the following program: 98 °C 30 s, (98 °C 10 s, 65 °C 50 s) × N, 65 °C 5 min. The cycle number N varies by sample and is determined by pilot PCR (quality check). PCR products were purified by DNA FragSelect XP Magnetic Beads (Smart Lifesciences) and sequenced in PE150 format on Illumina NovaSeq platform by Mingma Technologies Company.

### XR-seq

The original XR-seq libraries were prepared according to the established protocol (41) with minor modifications. Specifically, for (6–4)PP, purified excision products from 3.5 million cells were ligated to 2.5 pmol of each adapter in a 6-μl reaction, whereas purified excision products from 50 million cells were ligated to 35 pmol of each adapter in a 30-μl reaction for CPD. The ligation products were further purified by IP with corresponding DNA damage antibodies. Then, damage was repaired by the appropriate photolyase to enable PCR amplification. The produced libraries were gel purified and sequenced in PE150 format on Illumina NovaSeq platform by Mingma Technologies Company.

### Genome alignment and analysis

Since the excised oligonucleotides were as short as ∼26 nt, the 150-nt-long read 1 of the PE150 data was enough to cover excised oligonucleotides and poly(dA) tails. Therefore, only the data from read 1 were used for further analysis. For XR-seq, reads without 3′ adapter sequence were discarded, and 3′ adapter sequence was removed from the remaining reads by cutadapt (version 3.4) (47) with the command options -a TGGAATTCTCGGGTGCCAAGG -q 30 -j 8 --discard-untrimmed. Next, the adenine(s) at the 3′ end of processed reads were trimmed using custom Python scripts for a fair comparison between old and new methods.

For ATL-XR-seq, reads without a poly(dA) tail were discarded, and the poly(dA) tails of remaining reads were removed by cutadapt with the same command options -a "A{10}". Trimmed reads from both methods were aligned to hg38 human genome using bowtie2 (version 2.2.5) (48) with default settings. After mapping, aligned reads that were 21 to 31 nt in length were selected by executing the Linux commands and using samtools (version 1.7) (49), then deduplicated by Sambamba Markdup -r (version 0.8.1) (50), and converted to BED file format by bedtools (version 2.30.0) (51) for downstream analysis. Here, about 22 to 45 million filtered reads were retrieved from 45 to 87 million raw reads. Generally, 20 million filtered reads or 50 million raw reads are recommended for a typical ATL-XR-seq experiment. Custom Python scripts were used to retrieve sequences of 26-nt reads and calculate nucleotide and dinucleotide content of sequences. Histograms and scatterplots were generated using custom R scripts and R package ggplot2. Only reads aligned on the nuclear genome (chr1-22 and chrX) were used for calculation and analysis.

BigWig files of ATL-XR-seq and XR-seq were generated using function bamCoverage in deeptools (version 3.5.1) (52) with the parameters: --normalizeUsing RPKM (reads per kilobase per million mapped reads). Spearman correlation was computed by functions multiBigwigSummary and plotCorrelation in deeptools with the parameter: -bs 3000. Visualization of repair signals was implemented by IGV (53) with BigWig files. HeLa cells stranded RNA-seq BigWig files was downloaded from the ENCODE portal (accession: ENCFF344TQM), and Refseq genes were obtained from NCBI (https://s3.amazonaws.com/igv.org.genomes/hg38/ncbiRefSeq.sorted.txt.gz).

### A_n_-identifier deduplication and repair hotspots analysis

A_n_-identifier deduplication was performed by custom Python scripts, which counted the length of 3′-poly(dA) tails before removing them and then eliminated repeated reads based on their chromatin coordinates and the length of 3′-poly(dA) tails. Processed reads were converted to BED file format for downstream analysis.

Twenty-nucleotide chromatin sites on the genome were set up according to chromatin coordinates of aligned reads. The chromatin sites with reads per million mapped reads > 0.1 were defined as repair hotspots. The cutoff value can be adjusted based on the experimental conditions and sequencing depth. “Confident repair hotspots” were overlapping hotspots of two biological replicates. Reads per million mapped reads value (or RPKM) is commonly used for identifying “peaks” in ChIP-seq (54). However, compared with regular ChIP-seq, the distribution of repair is relatively random. Therefore, the numbers of hotspots are negatively correlated with the numbers of total qualified reads since more reads at a specific locus are required to reach the same threshold when the number of total qualified reads increases. The numbers of raw reads and qualified reads (after alignment and deduplication as described previously) can be found from deposited data.

Chromatin states combined segmentations of HeLa cells by chromHMM (55) were obtained from UCSC (https://genome.ucsc.edu/cgi-bin/hgTrackUi?g=wgEncodeAwgSegmentation&db=hg19). Analysis of confident repair hotspots on each chromatin state was based on the hg19 human genome.

### ATL-XR-qPCR assay

After the 1-h repair period, about six million UV-irradiated or control HeLa cells were resuspended in 140 μl of Tris-EDTA-NaCl-TritonX100 buffer (50 mM Tris-HCl pH 7.4, 2 mM EDTA, 150 mM NaCl, 1% Triton X-100) and incubated on ice for 20 min. The cell lysates were centrifuged at 20,000*g* for 1 h at 4 °C. The supernatants were transferred to a new 1.5-ml tube and incubated with 7 μl of Rnase A/T1 (Thermo Fisher Scientific) at 37 °C for 30 min, followed by the addition of 4 μl of proteinase K and incubation at 55 °C for 30 min. The DNA was then purified by phenol–chloroform extraction and ethanol precipitation, followed by purification with ssDNA/RNA Clean & Concentrator kit (ZYMO RESEARCH). Then, purified DNA samples were incubated with 0.5 μl of terminal deoxynucleotidyl transferase, 7.5 μl of 400 μM dATP, 5 μl of 10 × reaction buffer, and 5 μl of 2.5 mM CoCl_2_ in a total volume of 50 μl at 37 °C for 90 min. The reaction was inactivated at 75 °C for 20 min, followed by ethanol precipitation. Samples were divided into two portions (1/3 for (6–4)PP and 2/3 for CPD) and subjected to IP with damage-specific antibodies as described previously. Purified DNA samples were dissolved in 6.5 μl of 0.1× TE buffer and incubated with 1 μl of 10 μM Ad2-ATL and 7.5 μl of 2 × Instant Sticky-ends Ligase Master Mix at 4 °C overnight. After phenol–chloroform extraction and ethanol precipitation, the lesions in ligation products were reversed by (6–4)PP or CPD photolyase. As in ATL-XR-seq, a primer extension reaction was then performed.

The qPCR was performed on Roche LightCycler 480 System. Briefly, 1.25% (4.5 μl) of extended sample diluted in water was mixed well with 5 μl of 2× S6 Universal SYBR qPCR Mix (Novabio) and 0.5 μl of 10 μM primer mix (forward and reverse) in a total volume of 10 μl. The qPCR program was initial denaturation at 95 °C for 2 min, then denaturation at 95 °C for 15 s, and annealing and extension at 60 °C for 30 s for 45 PCR cycles. Raw data were normalized to the value of control data (Equation 1). Relative amount in Figure 6*B* is normalized data × 1000.

(1) $$Normalized\;data=\frac{1}{2^{Ct\;of\;UV}}-\frac{1}{2^{Ct\;of\;contol}}$$

## Data availability

The data reported in this paper have been deposited in the Sequence Read Archive (SRA) with accession number PRJNA804872. Scripts used in this paper are available at https://github.com/WooSZ/ATL-XR-seq_1.0.

## Supporting information

This article contains supporting information.

## Conflict of interest

The authors declare that they have no conflicts of interest with the contents of this article.
