# Supplementary material for: A new technique for genome-wide mapping of nucleotide excision repair without immunopurification of damaged DNA
Source: J Biol Chem. 2022 Mar 23;298(5):101863. doi: 10.1016/j.jbc.2022.101863 (PMC9034098; doi:10.1016/j.jbc.2022.101863)

Supplements to “A new technique for genome-wide mapping of nucleotide excision repair without immunopurification of damaged DNA”

Sizhong Wu[1], Yanchao Huang[1], Christopher P. Selby[2], Meng Gao[1], Aziz Sancar[2, *], and Jinchuan Hu[1, *]

[1]Shanghai Fifth People’s Hospital, Fudan University, and Shanghai Key Laboratory of Medical Epigenetics, International Co-laboratory of Medical Epigenetics and Metabolism (Ministry of Science and Technology), Institutes of Biomedical Sciences, Fudan University, Shanghai 200032, China; [2] Department of Biochemistry and Biophysics, School of Medicine, University of North Carolina, Chapel Hill, NC 27599-7260, USA.

To whom correspondence should be addressed. Email: hujinchuan@fudan.edu.cn; aziz_sancar@med.unc.edu.

Supplementary figures

Figure S1. Comparison of CPD repair analysis by ATL-XR-seq and XR-seq. Excision products were first purified by co-IP with repair factor (XPG) antibody in both methods in this study. However, they can also be purified by IP with damage antibodies at this step in XR-seq. Then, the simultaneous ligation of two adapters in XR-seq generates adapter dimers which cannot be completely eliminated by IP with CPD antibody. Therefore, an additional gel purification step is required in XR-seq. In contrast, sequential dA-tailing and adapter ligation does not produce any dimers, thus the CPD-IP and gel purification steps can be omitted in ATL-XR-seq. Unique steps of each method are shown in blue (ATL-XR-seq) and red (XR-seq).

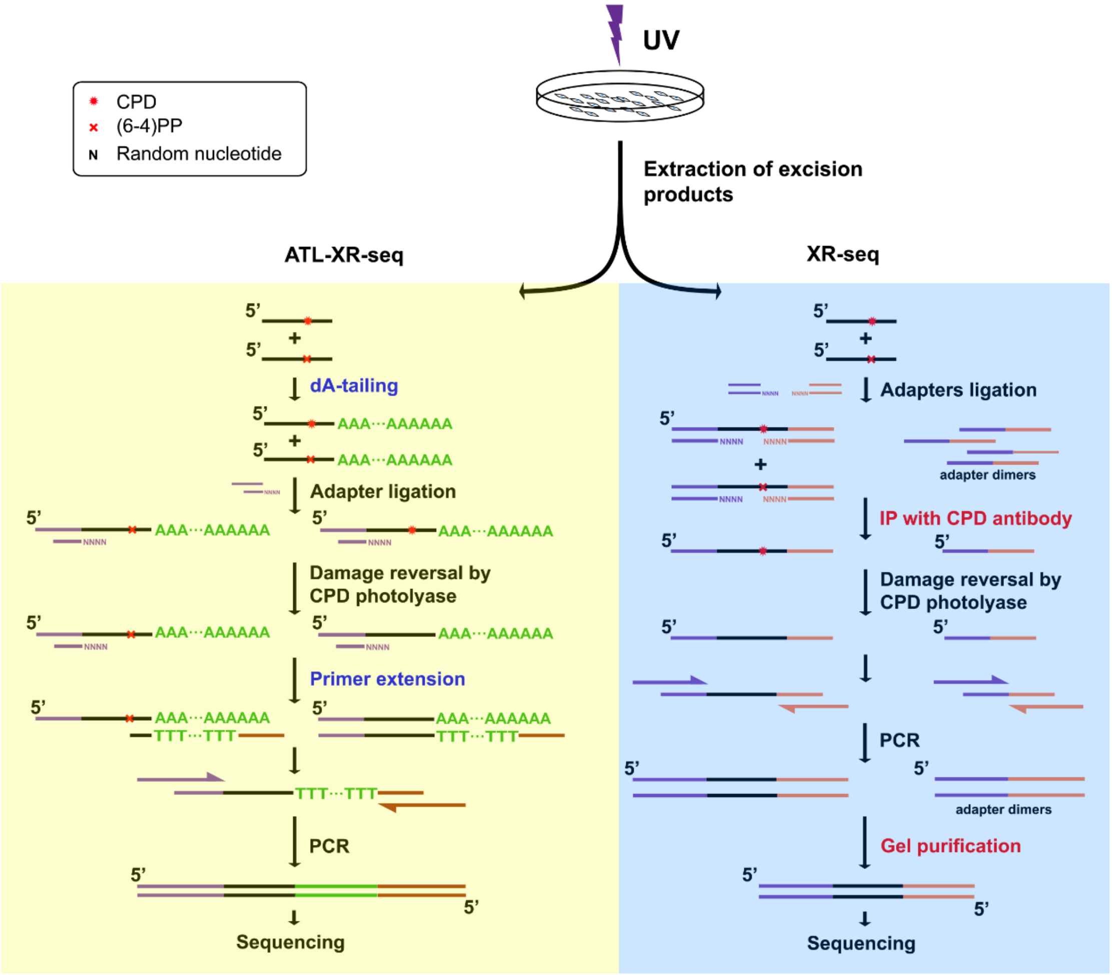

Figure S2. Length of poly(dA)-tails. *A*, Titration of dA tailing reaction components using a FAM-labeled 26 nt substrate (5FAM-26nt). The products were separated by 8% denaturing polyacrylamide gel electrophoresis, and the gel was visualized by fluorescence imaging on Tanon 3500B Gel Imaging System. Markers (lane 1) were loaded onto the same gel and visualized by Syber Gold staining. *B* and *C*, Length distribution of poly(dA)-tails of (6-4)PP (*B*) and CPD (*C*) excision products determined by ATL-XR-seq.

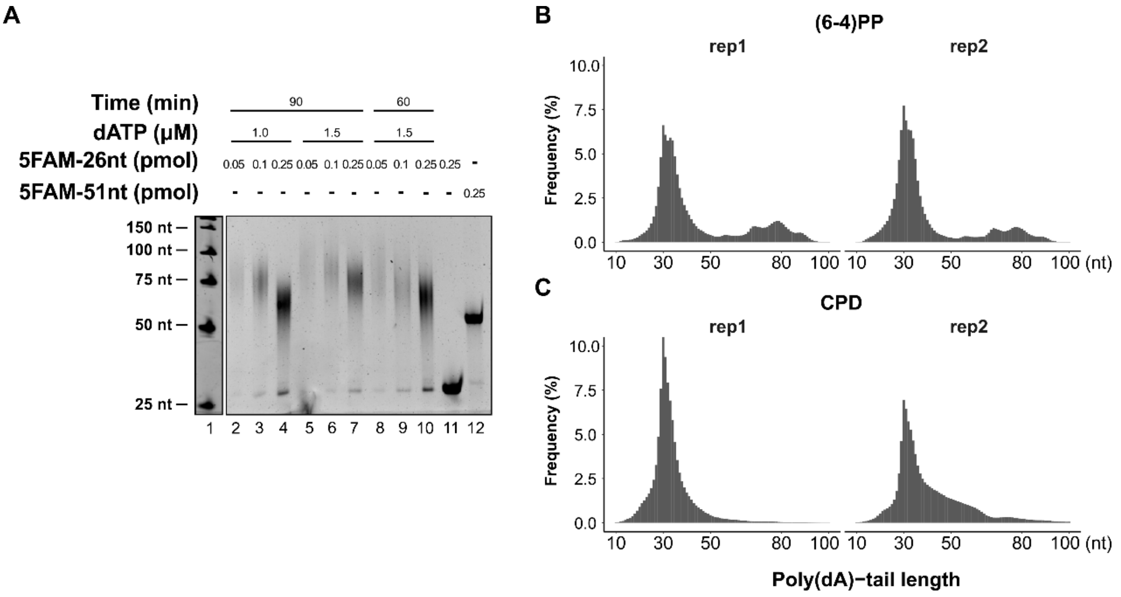

Figure S3. Length distributions and nucleotide frequencies of ATL-XR-seq reads. *A-F*, Similar to Figure 2, *B-G* except replicate 2 is shown here. Results are shown for repair of (6-4)PP (*A-C*) and CPD (*D-F*).

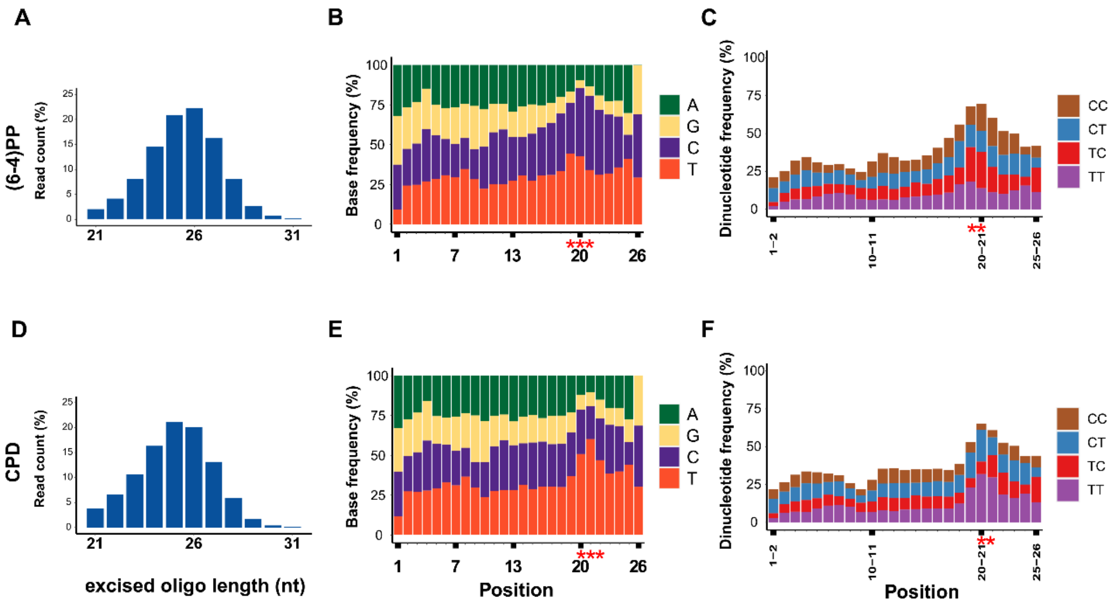

Figure S4. Repair pattern along genes. *A*, Similar to Figure 4*A* except that the repair patterns by XR-seq and both replicates of ATL-XR-seq in a 3.72-Mb region on chromosome 13 (containing the 1.36-Mb region in Figure 4*A*) are shown. *B* and *C*, Similar as Figure 4, *B* and *C* except that replicate 2 of ATL-XR-seq is shown.

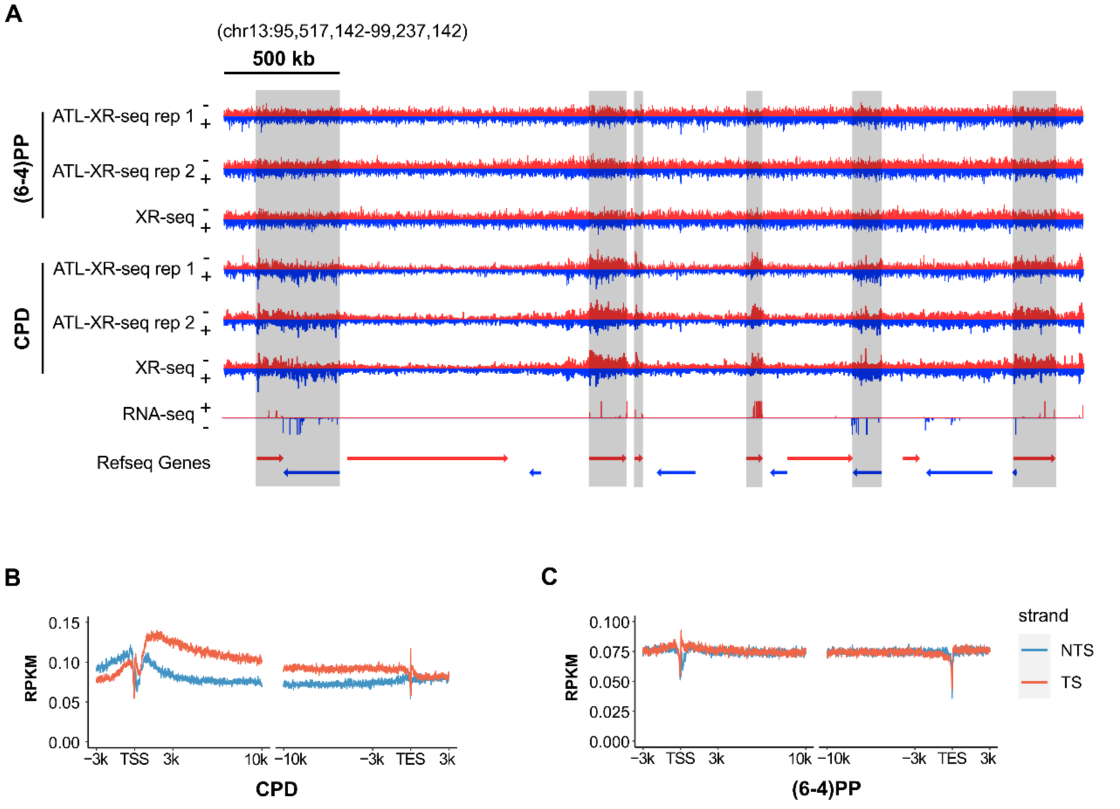

Figure S5. Quantification of (6-4)PP and CPD repair products by ATL-XR-qPCR. Plotted as in Figure 6*C* except that replicate 2 of ATL-XR-qPCR is shown.

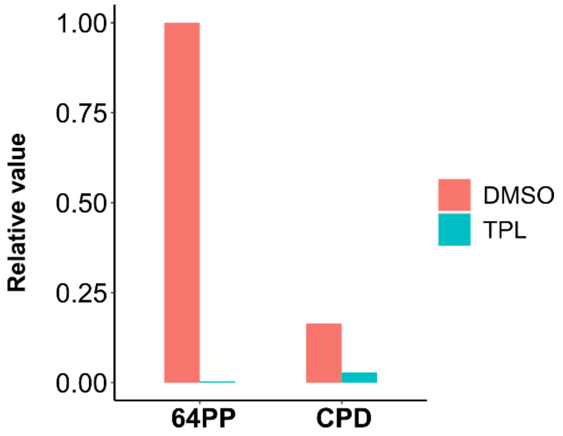

Supplement: Supplemental Figures S1–S5 [file mmc1.pdf]
